# Health Risk Assessment of Nitrate in Drinking Water with Potential Source Identification: A Case Study in Almaty, Kazakhstan

**DOI:** 10.3390/ijerph21010055

**Published:** 2023-12-30

**Authors:** Yerbolat Sailaukhanuly, Seitkhan Azat, Makhabbat Kunarbekova, Adylkhan Tovassarov, Kainaubek Toshtay, Zhandos Tauanov, Lars Carlsen, Ronny Berndtsson

**Affiliations:** 1 Laboratory of Engineering Profile, Satbayev University, 22a Satpaev Str., Almaty 050013, Kazakhstan; s.azat@satbayev.university (S.A.); m.tatibayeva@stud.satbayev.university (M.K.); 2 Central Asian Institute for Ecological Research, 300/26 Dostyk Ave., Almaty 050012, Kazakhstan; adil@asianecology.kz; 3 Faculty of Chemistry and Chemical Technology, Al-Farabi Kazakh National University, 71 Al-Farabi Ave., Almaty 050040, Kazakhstan; kainaubek.toshtay@kaznu.kz (K.T.); zhtauanov@nu.edu.kz (Z.T.); 4 Awareness Center, Linkøpingvej 35, Trekroner, DK-4000 Roskilde, Denmark; lc@awarenesscenter.dk; 5 Division of Water Resources Engineering & Centre for Advanced Middle Eastern Studies, Lund University, P.O. Box 118, SE-221 00 Lund, Sweden

**Keywords:** drinking water, groundwater, nitrate pollution, health risk, infant health, SDGs

## Abstract

Infant mortality in Kazakhstan is six times higher compared with the EU. There are several reasons for this, but a partial reason might be that less than 30% of Kazakhstan’s population has access to safe water and sanitation and more than 57% uses polluted groundwater from wells that do not comply with international standards. For example, nitrate pollution in surface and groundwater continues to increase due to intensified agriculture and the discharge of untreated wastewater, causing concerns regarding environmental and human health. For this reason, drinking water samples were collected from the water supply distribution network in eight districts of Almaty, Kazakhstan, and water quality constituents, including nitrate, were analyzed. In several districts, the nitrate concentration was above the WHO and Kazakhstan’s maximum permissible limits for drinking water. The spatial distribution of high nitrate concentration in drinking water was shown to be strongly correlated with areas that are supplied with groundwater, whereas areas with lower nitrate levels are supplied with surface water sources. Based on source identification, it was shown that groundwater is likely polluted by mainly domestic wastewater. The health risk for infants, children, teenagers, and adults was assessed based on chronic daily intake, and the hazard quotient (HQ) of nitrate intake from drinking water was determined. The non-carcinogenic risks increased in the following manner: adult < teenager < child < infant. For infants and children, the HQ was greater than the acceptable level and higher than that of other age groups, thus pointing to infants and children as the most vulnerable age group due to drinking water intake in the study area. Different water management options are suggested to improve the health situation of the population now drinking nitrate-polluted groundwater.

## 1. Introduction

Access to safe drinking water supply is an important UN Sustainable Development Goal (SDG). Kazakhstani authorities pay significant attention to water management policy and, as such, to SDG 6 (Clean Water and Sanitation) [1]. However, the demand for water resources in Kazakhstan is very high, and water quantity and quality continue to decrease [2]. The importance of SDG 6 cannot be underestimated because, in 2020, safe drinking water was only available to 74% of the world’s population [3]. Furthermore, water stress, although below 20% globally, is high, being >75% in Southern and Central Asia [3]. 

The link between poor sanitation and health is well-established [1]. There is a lack of coordination among the different institutions responsible for different types of water infrastructure and a lack of information sharing and exchange, particularly for monitoring data [1]. Water supply must be seen as a combination of sanitation and hygiene promotion [1,4]. Many contaminants in groundwater, such as widespread nitrate (NO_3_^−^) and fluoride pollutants, are suspected to affect human health by causing, e.g., colon, rectum, ovarian, bladder, gastrointestinal, and oral cancer and methemoglobinemia [5,6,7,8,9,10,11,12,13,14,15], in addition to increasing the risk of congenital anomalies [12]. A maximum reference dose of 10 mg/L nitrate for formula-fed infants, older children, and adults has been established [16]. Under normal conditions, nitrate levels in groundwater do not exceed 10 mg/L. Nitrate levels above 10 mg/L in groundwater are an indicator of its anthropogenic effects on water quality [17]. Nitrate contamination can be exacerbated by several anthropogenic factors, such as nitrogen fertilizers, septic tank leaks, waste dumps, sewage discharges, contaminated land, unhygienic sanitation practices, and animal waste [18,19,20,21]. The WHO has established that nitrate-rich and oxygen-poor drinking water stagnating in galvanized steel pipes can form nitrite in water networks where Nitrosomonas bacteria are present or where chlorination disinfection is used [22]. Furthermore, it has been found that nitrification can occur and nitrate and nitrite levels in drinking water can rise when a distribution system is overloaded with excess free ammonia. Nitrates and nitrites can also form due to nitrification in the water supply or distribution system [22]. 

More than 94% of the urban population of Kazakhstan has a centralized water supply; in rural areas, the amount is 84.4% [23]. However, citizens in several regions often complain about the quality of drinking water, and in some areas, certain disease patterns are present [4]. It should be noted that Kazakhstan’s drinking water quality standards differ from those of other OECD countries due to the lack of regulation of several chemicals in drinking water [24]. Despite a decrease in manufacturing in Kazakhstan, pollution levels in cities and industrial centers are very high [25]. Most of Kazakhstan’s water supply and water management system has been in place since the Soviet era. Physically outdated, it is in great need of modernization [26]. As pointed out by Vodokanal Invest Consulting [26], the deterioration of the water supply, sanitation network, and facilities is responsible for a significant part of urban water supply and sanitation problems, which means that its dependability and continuity are not assured. The destruction of the antirust coating in pipes results in the secondary contamination of drinking water in the water supply network. The quality standards of water utility services are based on the rules that were established in the former Soviet Union or are almost identical to current Russian standards (deterioration of the water supply, sanitation network, and facilities) [24]. The contamination of drinking water sources, combined with the inefficient operation of treatment plants, has resulted in a general decrease in drinking water quality [4,26]. 

Kazakhstan’s infant mortality rate is one of the highest among the Central Asian states, with about 18 deaths per 1000 live births. This is about six times the average rate for the EU. At the same time, less than 30% of Kazakhstan’s population has access to safe water and sanitation, and more than 57% uses groundwater that does not comply with international standards. High nitrate content in surface and groundwater is causing concern over environmental and human health. Previous studies have shown that nitrate may constitute a major groundwater pollutant at various locations in Kazakhstan [27]. Urban and industrial pollution levels remain high despite a decrease in manufacturing, possibly due to wastewater discharge or the heavy use of fertilizers [28]. However, there are still only a few comprehensive studies on groundwater and tap water quality in the region. In view of this, the main objective of this study was to improve the knowledge regarding health risks posed by nitrate in groundwater. There is an urgent need to decipher the potential health effects of polluted groundwater in Kazakhstan and to suggest improved water quality management. Thus, we assessed the general drinking water quality of municipal water in Almaty, Kazakhstan, which is groundwater. Furthermore, we determined the non-carcinogenic risk to human health posed by the nitrate content of drinking water in specified age ranges, i.e., infants, children, teenagers, and adults. Based on the results, we discuss source identification and suggest measures that decision-makers and water managers need to consider to improve drinking water quality in Almaty and other regions of Kazakhstan. The results of this study are crucial for developing contaminated drinking water treatment strategies in the study area and other regions with similar socioeconomic environments and groundwater conditions.

## 2. Materials and Methods

### 2.1. Study Area 

**Figure 1:**
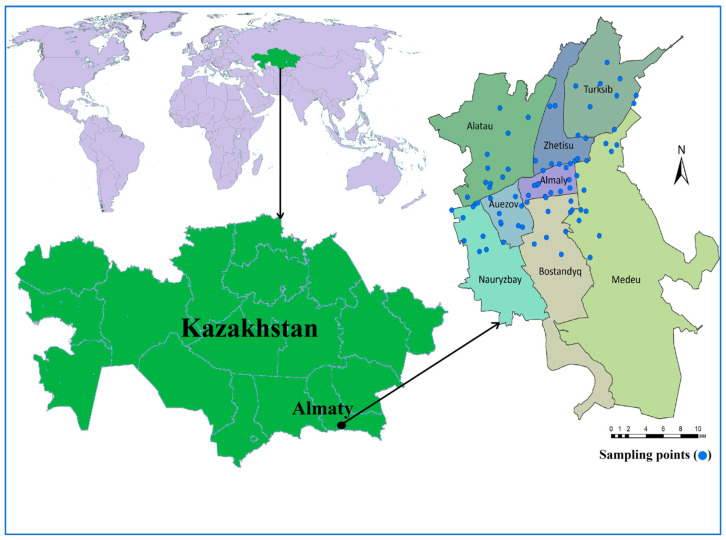
Almaty City in Kazakhstan and the experimental study area with sampling locations for the domestic tap water supply.

The study area was Almaty City, the former capital of Kazakhstan, in the southeastern part of the country between 43°16′39″ north latitude and 76°53′45″ east longitude (Figure 1). It consists of eight districts (Alatau, Almaty, Auezov, Bostandyq, Medeu, Nauryzbay Turksib, and Zhetisu). The area is located about 500 m above mean sea level, with an average monthly temperature in the summer of +23.8 °C and in the winter of −4.7 °C. The average precipitation ranges from 298 mm to 1013 mm per year [29,30]. According to a survey conducted in 2022, the population is over 2 million [31]. Several sources supply drinking water to Almaty City, including both surface water and groundwater. Several 150–500 m deep wells are used to extract about 70% of the total water supply [29]. Groundwater sources supply the northern and northwestern parts of the city (Alatau, Auezov, Nauryzbay, Turksib, and Zhetisu districts), whereas surface waters are provided to the central, southern, and southeastern parts (Almaty, Bostandyq, and Medeu districts) [29] (Figure 1). Currently, the extraction and intake of water are carried out from groundwater sources from the Almaty, Maloalmatinsky, and Talgar aquifers. Depending on the depth of the aquifer, the wells at the Almaty and Talgar water supplies are operated by three groundwater areas with different depths: soil surface to 150 m, 50–300 m, and 300–500 m. About 170 wells operate daily, and a total of 386 artesian wells have a 1,092,000 m^3^/day water intake capacity [29]. The surface water sources include the Aksay, Bolshaya Almatinka, Malaya Almatinka, Kargaly, and Kim-Asar rivers [27]. 

According to [31], the city’s water consumption was 172,000,000 m^3^ in 2021. The total length of the Almaty water supply network and pipelines is about 3700 km. To improve the water supply, 170 km of water pipelines were built in 24 smaller districts in the Nauryzbay, Alatau, and Turksib districts of Almaty in 2021 (36,000 homes) [29]. 

Before reaching city residents, water goes through the following stages of purification for river water: preliminary sedimentation, coagulation, filtration, and water disinfection. Preliminary settling takes place in open-type structures (radial settling tanks, settling ladles, daily regulation pools). Coagulation is carried out using coagulants, such as ferric chloride and aluminum sulfate. To improve flocculation, a flocculant, polyacrylamide, is added. Water that has undergone coagulation and clarification in settling tanks of various types is collected at filter stations. Filtration takes place using rapid filter media, such as finely crushed expanded clay. Next, sodium hypochlorite, obtained with the electrolysis of ordinary table salt, is used to disinfect the water [29]. Groundwater is mainly filtered and disinfected before being delivered to consumers. However, these treatment processes are not effective for nitrate removal because nitrate is stable and highly soluble with a low potential for co-precipitation and adsorption.

Water and wastewater service infrastructure has not been properly maintained for decades [25,27]. As a result, the existing infrastructure needs renovation. The surveying authorities [32] have assessed that most of the water supply networks in the major cities, surface water treatment plants, and mechanical–biological wastewater treatment plants need renovation [25]. This is, however, an immense work. In 2021, the total length of the sewerage network in Almaty was 1868 km, of which 1097 km needed to be replaced. There are 29 pumping stations for wastewater disposal, of which more than half need replacement. The sewer networks have been in operation for more than 70 years, and they now have a wear rate of 59%. The sewage treatment facilities have a wear rate of 60% [33]. 

### 2.2. Sampling and Analyses 

In total, 80 tap water samples were collected in November 2022. The sampling was performed in a spatial and temporal representative manner. The water samples were taken close to major distribution points to reflect larger delivery areas and during a time of day to reflect mean daily flow. November was chosen as the sampling time between the dry and wet periods, thus representing the average groundwater levels during the year. The 0.5 L tap water samples were stored in plastic containers at a temperature of 4 °C. Before filling, the containers were cleaned and rinsed several times with tap water. A label was then attached with the code, location, and sampling date. Different physicochemical parameters such as pH, electrical conductivity (EC), total dissolved solids (TDSs), concentrations of cations (Ca^2+^, Mg^2+^, K^+^, and Na^+^), and anions (NO_3_^−^, NO_2_^−^, SO_4_^2−^, and Cl^−^) were determined after filtering through a 0.45 μm filter. EC, TDS, and pH were measured immediately after sampling using a handheld multi-parameter water quality analyzer HI2030-02 (Hanna Instruments, Cluj-Napoca, Romania) and pH meter (pH-150MI, Moscow, Russia). The capillary electrophoresis technique (Kapel-150M, Lumex, Russia) was used to analyze cations and anions following the standards PNDF 14.1:2:4.167-2000 [34] and PNDF 14.1:2:4.157-99 [35]. The collected data were used to identify potential contamination sources in the studied area. 

### 2.3. Health Risk Assessment 

A health risk assessment is a key step in risk management and a crucial part of the final decision-making process. A health risk assessment is one of the most efficient approaches for determining the risk to human health from environmental contaminants. In this study, direct drinking water ingestion was focused on as the main pathway of exposure. The human health risk assessment methodology of USEPA [36] was used to evaluate the non-carcinogenic risks due to the intake of drinking water contaminated with nitrate. The assessment was performed for infants (0–2 years), children (2–6 years), teenagers (6–16 years), and adults (≥16 years). It should be noted that according to USEPA [16], nitrate is considered a non-carcinogenic risk parameter for human health. In the exposure assessment, the chronic daily intake (CDI) (mg/kg/day) of nitrate ingested from drinking water was calculated using [36]:

(1) $$CDI=\frac{C\times IR\times ED\times EF}{BW\times AET}$$

The parameters in this equation that were used to calculate the CDI and the hazard quotient (HQ; Equation (2)) are shown in Table 1.

When the exposure dose of a contaminant is higher than the reference dose, which is usually expressed as the HQ, toxic effects are likely to occur:

(2) $$HQ=\frac{CDI}{RfD}$$

where RfD is the (reference dose) of nitrate exposure and a measure of chronic non-carcinogenic risks (1.6 mg/kg/day; [16]). When HQ is greater than 1, the non-carcinogenic risk is greater than the acceptable level, indicating a potential health risk [36]. 

## 3. Results and Discussion

### 3.1. Physicochemical Parameters 

To check the analytical accuracy between the concentrations of total cations (TC^+^: Ca^2+^, Mg^2+^, Na^+^, and K^+^) and total anions (TA^−^: Cl^−^, SO_4_^2−^, and NO_3_^−^) (meq/L) in each sample, the charge balance error (CBE) was calculated as:

(3) $$CBE=\frac{\sum {TC}^{+}-\sum {TA}^{-}}{\sum {TC}^{+}+\sum {TA}^{-}}$$

This indicated that the calculated CBE was within the acceptable limit of ±10% for all constituents. The tap water analysis results were compared to the World Health Organization [11] and Kazakhstan’s maximum permissible limits (MPLs) for drinking water [24] (Table 2). Table 2 shows that pH ranged from 6.03 to 8.25 with a mean of 7.42, indicating that the water is slightly acidic to alkaline. EC ranged from 62 to 993 µS/cm with a mean of 397 µS/cm, indicating a high variability in ion concentration in the study area’s drinking water. According to [11], drinking water with a TDS less than 600 mg/L is generally considered to be of good quality, while a concentration greater than 1000 mg/L is unacceptable for drinking purposes. In this study, the TDS concentration in the drinking water samples ranged from 31 to 323 with a mean of 203 mg/L. All samples were within the MPL limits (Table 2); thus, the drinking water in the study area had concentrations of pH, EC, and TDS well below the MPL for drinking water. 

The mean concentration of cations was in the order Ca^2+^ (42.6 mg/L) > Na^+^ (11.5 mg/L) > Mg^2+^ (8.01 mg/L) > K^+^ (1.54 mg/L), whereas anions were in the order SO_4_^2−^ (29.7 mg/L) > NO_3_^−^ (16.5 mg/L) > Cl^−^ (9.85 mg/L) > NO_2_^−^ (not detected). As shown in Table 2, the concentration of cations and anions in all drinking water samples was lower than the MPL for drinking water [11,24] except for nitrate (NO_3_^−^). Nitrite was not detected in any sample. In oxygenated water systems, nitrate is more stable than nitrite due to the latter’s higher reactivity [22]. The concentration of cations in the drinking water was below the WHO [11] and Kazakhstan MPL [24] for drinking water (Table 2). The concentration of anions (SO_4_^2−^, Cl^−^) in the samples was also well below the Kazakhstan MPL [24]. However, the nitrate concentrations were in the range from 2.23 to 59.8, with a mean of 16.5 mg/L, and sometimes exceeded both the WHO and Kazakhstani MPL. Thus, according to our definitions, the groundwater is affected by humans, as seen from the mean value of nitrate equal to 16.5 mg/L (>10 mg/L), and it is occasionally polluted by nitrate (>KZ MPL = 45 mg/L and >WHO MPL = 50 mg/L).

### 3.2. Spatial Distribution of Nitrate 

**Figure 2:**
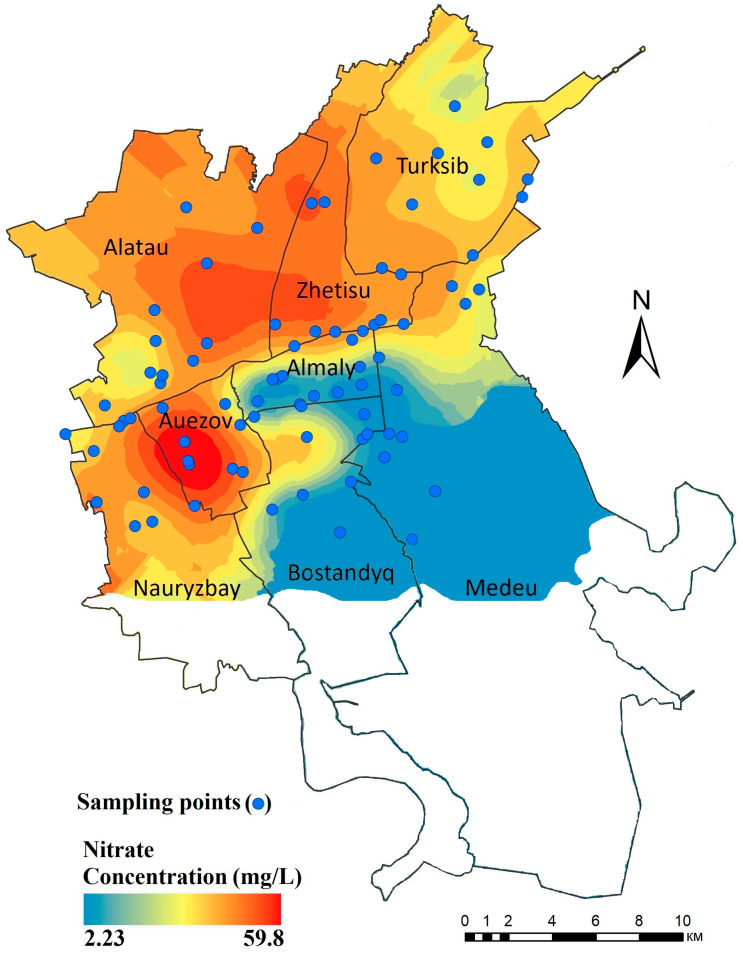
Spatial distribution of nitrate concentration in drinking water in the study area.

Due to the elevated concentrations of nitrate in the drinking water, a specific study was performed on the spatial variation in nitrate concentration. Figure 2 shows the spatial distribution of nitrate in the tap water, with higher concentration areas in red and lower concentration areas in blue. The spatial distribution shows that high concentrations were observed in the Auezov, Zhetisu, Alatau, and Nauryzbay districts and a few patches in the Turksib and Bostandyq districts, as shown in Figure 2. This suggests that nitrate concentrations in these districts are high, thus increasing the health risk from ingesting nitrate in these areas. Figure 2 shows that the spatial distribution of nitrate concentration correlates well with the type of drinking water source in Almaty. Areas with elevated levels of nitrate correspond to districts supplied with groundwater sources, whereas districts provided with surface water sources show lower levels of nitrate. According to the municipal water service [29], groundwater sources are used in the northern and northwestern parts of the city (Alatau, Auezov, Nauryzbay, Turksib, and Zhetisu districts), whereas surface water is supplied to the central, southern, and southeastern parts (Almaty, Bostandyq, and Medeu districts). Two water samples in the Auezov district with nitrate concentrations of 59.8 mg/L and 57.2 mg/L exceeded both the Kazakhstan (45 mg/L) and the WHO MPL (50 mg/L). However, other samples were lower than both MPLs (Table 2). Anthropogenic sources and activities, such as agricultural fertilizer usage, septic system and sewage leaks, and human and animal waste, contribute to an enrichment in nitrate concentration in groundwater [39,40,41]. 

A strong correlation between nitrate and other anions (such as chloride, phosphate, and sulfate) was found in earlier studies [42], suggesting that these pollutants come from the same source such as sewage water leaks into the groundwater. Elevated nitrate concentrations in tap water could be related to domestic sewage due to the poor condition of water pipes and insufficient wastewater treatment [40,43]. The poor state of the sewage network and the leaching of domestic effluents and sewage into groundwater might be the major causes of nitrate contamination in Almaty. Mester et al. [19] reported that domestic wastewater is one of the most significant sources of nitrogen compounds. Due to high mobility and low environmental retention, nitrate can easily infiltrate groundwater sources and contaminate shallow aquifers [21,44,45]. Also, deeper groundwater aquifers may be affected due to drawdown of the groundwater table. Previous studies in the region have shown that the drawdown of the groundwater table may be up to 80 m with a cone of depression area of up to 150 km^2^ [46]. This facilitates the transport of shallow pollutants to deeper groundwater levels.

Furthermore, nitrate contamination may also be caused by inadequate sanitation facilities and infrastructure [43]. Due to rainfall infiltration, groundwater can become contaminated with nitrate from domestic effluents and sewage waste [45]. The intensive use of fertilizers [28] may also cause nitrate contamination. However, agricultural activity in the study area is not much developed. Consequently, better management of sanitation facilities is critical to reduce nitrate concentrations in the groundwater [47]. Improving and expanding sewage collection networks and wastewater treatment systems should be prioritized to improve groundwater quality [42,47,48]. It has been observed that specific districts of Almaty (Alatau, Auezov, and Nauryzbay) have high concentrations of nitrate where local inhabitants have constructed improper sewage tanks that leak into the soil and contaminate the groundwater [27].

### 3.3. Source Identification of Nitrate 

**Figure 3:**
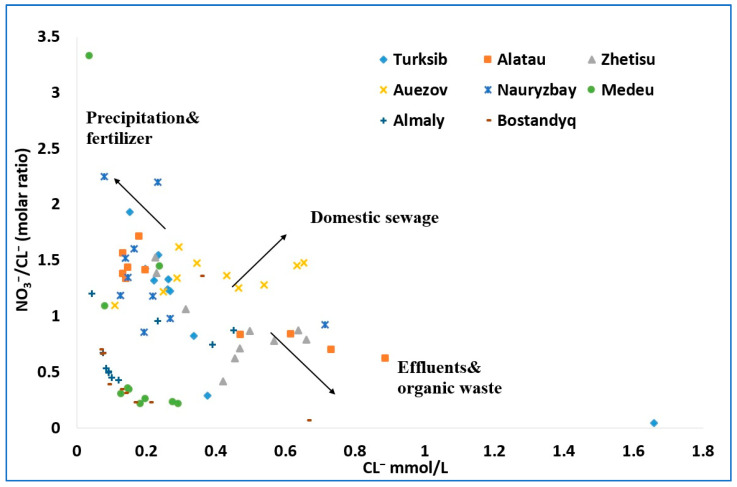
NO_3_^−^/Cl^−^ molar ratio variation in drinking water samples as a function of the amount of Cl^−^ ions.

Understanding the level and origin of nitrate (NO_3_^−^) pollution is crucial in managing nitrogen health risks and advancing sustainable water management [49]. Zhang et al. [50] reported that nitrogen fertilizers, industrial discharge, municipal wastewater, animal waste, soil organic matter, and atmospheric nitrogen deposition are all potential causes of nitrate pollution in water. Numerous studies [18,20,51,52] have used stable isotope analysis, such as dual nitrogen and oxygen isotope ratios of nitrate, to identify nitrate pollution sources. Also, the sources of nitrate contamination in water have been found using relationships between nitrate, nitrite, ammonium, and chloride [42,53]. According to Liu et al. [54], chloride is not affected by physicochemical or microbiological processes that take place in groundwater, making it useful as a sewage impact indicator. Natural sources (mineral dissolution), agricultural chemicals, septic effluents, and road salt are potential sources of Cl^−^ [54]. In practice, nitrate and chloride ions are highly related to human activities that are mostly of organic origin [41]. The examination of the NO_3_^−^/Cl^−^ vs. Cl^−^ ratio in groundwater may thus represent the human impact on the groundwater content and can be used to identify the various sources of nitrate in drinking water [17,54]. This method compares the NO_3_^−^/Cl^−^ concentration ratios with the assumption that Cl^−^ is scarcely transformed in the environment [41,54]. This characteristic suggests that when Cl^−^ is higher than the NO_3_^−^/Cl^−^ ratio, nitrate pollution is related to domestic effluent and sewage, whereas reverse ratios are typically related to precipitation/fertilizers [54]. The approach assumes that Cl^−^ in the wastewater does not affect the NO_3_^−^/Cl^−^ vs. Cl^−^ ratio in groundwater to a great extent. Figure 3 shows the output of these calculations. High concentration ratios of NO_3_^−^/Cl^−^ and Cl^−^ are seen in water samples in the Auezov district, indicating that nitrate has a domestic sewage origin [17]. Drinking water samples from the Alatau, Almaty, Auezov, Nauryzbay, and Turksib districts have higher concentration ratios of NO_3_^−^/Cl^−^ and lower Cl^−^, demonstrating that precipitation and fertilizers may be the primary sources of nitrate [17]. However, nitrate in the Alatau, Auezov, and Zhetisu districts may be from effluents and organic waste sources. Although the primary sources of nitrate in the Alatau, Almaty, Auezov, Nauryzbay, and Turksib districts are precipitation and fertilizers, some samples may have been influenced by the discharge of effluents and organic waste in the Alatau, Auezov, and Zhetisu districts. These observations correspond to the districts of Almaty (Alatau, Auezov, and Nauryzbay) with high concentrations of nitrate where domestic sewage and organic waste from local inhabitants contaminate groundwater sources. 

### 3.4. Health Risk Assessment 

Nitrate in drinking water may be harmful to human health in any amount [14]; therefore, to evaluate the non-carcinogenic risk of nitrate in drinking water, the daily intake should be calculated. Thus, human health risk was calculated according to the USEPA [38] methodology. The results of the evaluation of non-carcinogenic risks (HQ) for infants, children, teenagers, and adults are presented in Table 3. The HQ for infants, children, teenagers, and adults ranged from 0.07 to 1.95, 0.06 to 1.50, 0.05 to 1.26, and 0.04 to 1.12 with a mean of 0.54, 0.41, 0.35, and 0.31, respectively (Table 3). However, the USEPA has set a human health concern limit of 1 per element for the HQ [38]. Non-carcinogenic health risks increased in the following order: adults < teenagers < children < infants. When HQ > 1, the non-carcinogenic risk is greater than the acceptable level, indicating a potential health risk [36]. High health risk is in line with the sampling points of drinking water with high nitrate concentrations. Under these conditions, drinking water contaminated with nitrate may pose a serious health risk if the municipal authority takes no action to reduce nitrate contamination. 

Table 3 shows that most HQ values for adults and teenagers are acceptable, except for those in one district (Auezov). This means that there is an adverse health risk of nitrate in drinking water for adults and teens. Some HQs for children and infants are above acceptable levels in two (Auezov and Nauryzbay) and four (Auezov, Alatau, Zhetisu, and Nauryzbay) districts, respectively. Unsurprisingly, children and infants are at higher non-carcinogenic risk from nitrate in drinking water than adults and teenagers due to their lower body weight and relatively immature enzyme metabolism compared with adults and teenagers [22]. Table 3 demonstrates that the infant and child population of the Auezov district is at higher health risk from nitrate-contaminated water than the rest of the study area. 

In the study area, there were 21 samples (26%) with higher HQs than the acceptable levels, indicating that these locations may have negative effects and pose a health risk to infants. According to earlier studies [5], ingested nitrate leads to the conversion of nitrite with hemoglobin into methemoglobin (MetHb). The transformation of nitrate into nitrite occurs primarily in the digestive tract via enteric bacteria. Therefore, not only the dose but also the type and number of bacteria can play a role in the risk of nitrate-induced methemoglobinemia [5]. According to the WHO [22], about 5% of nitrate in healthy adults is metabolized into nitrite. Nitrate can also be metabolized in the stomach into nitrite when the pH of the stomach liquid is high (above pH 5) [5,22]. This is an issue for adults who suffer from gastrointestinal disorders and achlorhydria. Still, it is also an issue for infants because the pH of their gastrointestinal systems is high enough to encourage bacterial growth. Thus, it is generally considered that infants are the most susceptible age group to nitrate-induced methemoglobinemia, especially those between 0 and 3 months of age [16]. In addition, adults who consume nitrate-containing water may develop high blood pressure, stomach cancer, thyroid, and respiratory disorders [40,45,55]. Due to their high thyroid hormone turnover and limited intrathyroidal reserves throughout fetal and early life, pregnant women and babies are the most susceptible groups. Nitrate overdose might be dangerous for pregnant women and increase the chance of congenital disabilities [22]. 

The health and water management authorities of Almaty need to take necessary actions to reduce the nitrate content in districts where health risks of nitrate were found. This can be achieved by prioritizing these sections of water supplies during the renovation of water pipes and treatment plants. The authorities also need to consider if it is necessary to exchange parts of the groundwater supply for cleaner surface water. Ultimately, the drinking water supply needs to be treated for nitrate before consumption.

## 4. Conclusions

The problem of drinking water pollution and the causes of the increase in and occurrence of nitrate in drinking water were studied in eight districts of Almaty, Kazakhstan. An analysis of physicochemical parameters (pH, EC, TDS, cations, and anions) of collected drinking water samples revealed that most of the physicochemical water quality parameters are well below the MPL in Kazakhstan, except for nitrate. The spatial distribution of nitrate concentrations disclosed that hot spots of high concentrations occur in the Auezov, Zhetisu, Alatau, and Nauryzbay districts and a few patches in the Turksib and Bostandyq districts. The results indicate that the non-cancer risk associated with nitrate is higher in these districts. The spatial distribution of high nitrate concentrations correlated well with districts that are supplied with groundwater. Districts supplied with surface water showed low levels of nitrate in tap water. To explain these findings, chemical ion ratios, such as NO_3_^−^/Cl^−^ vs. Cl^−^, were studied, as these may represent anthropogenic impact and can be used to identify potential nitrate sources. As a result, we found that nitrate in drinking water likely comes from domestic sewage, organic waste, and precipitation, respectively. The high concentrations of nitrate in water in the Auezov district are likely of domestic sewage origin. The presence of nitrate in the Alatau, Almaty, Auezov, Nauryzbay, and Turksib districts is likely due to precipitation and fertilizers, whereas the discharge of effluents and organic waste may also influence the nitrate level in the Alatau, Auezov, and Zhetisu districts. These observations correspond to the communities of Almaty (Alatau, Auezov, and Nauryzbay) with high concentrations of nitrate where domestic sewage and organic waste may contaminate the groundwater. 

The health risk due to nitrate in drinking water was found to increase in the order adult < teenager < child < infant. Thus, children and infants are at higher risk from nitrate-contaminated water in Auezov, Alatau, Zhetisu, and Nauryzbay districts. Consequently, it can be concluded that children and infants are more likely to be exposed to non-carcinogenic health risks from nitrate in drinking water. This depends on their lower body weight and relatively immature enzyme metabolism compared with adults and teenagers. The infant mortality rate in Kazakhstan is about six times higher compared with the EU [56,57]. Polluted groundwater is probably a contributing factor to this problem. Less than 30% of the Kazakhstani population has access to safe water, and about 57% uses groundwater from wells. Similarly, about 50% of the Kazakhstani population drinks water that does not comply with international standards. Despite this, there are only a few studies on the relationship between groundwater and tap water quality in the region. This paper tried to fill some of this knowledge gap. Nitrate pollution is likely not the only factor responsible for the increased child mortality rate, and there is a need for improved knowledge in this regard. 

Appropriate and sustainable drinking water management is important to mitigate the risk of unwanted and harmful effects due to nitrate and other kinds of pollutants [58]. Thus, further studies need to elucidate possible long-term impacts, especially regarding deeper groundwater aquifers. It is also necessary to raise public awareness of the harmful effects of pollutants in groundwater and how they translate to the drinking water supply. Further, establishing a drinking water monitoring network as early as possible is recommended to determine the long-term impact. The information gathered from water quality monitoring should additionally be used to inform relevant stakeholders. The results of the current study can give local experts and decision-makers a better understanding of the drinking water hazards. In the short term, residents should be provided with drinking water filters to reduce the amount of nitrate. In the long term, other treatment possibilities are at hand such as membrane technologies, oxidation, and biological processes. This will help mitigate the risk to public health and ensure safe drinking water in these areas.

## Figures and Tables

**Table 1:** Parameters used to calculate the chronic daily intake (CDI) and the hazard quotient (HQ) for nitrate in drinking water.

No.	Parameter	Unit	Infant (0 ≤ 2 Years)	Child (2 ≤ 6 Years)	Teenager (6 ≤ 16 Years)	Adult (≥16 Years)	Reference
1	C, concentration	mg/L	In present study				
2	IR, ingestion rate	L/day	0.62	0.78	2.0	2.5	[37]
3	ED, exposure duration	year	1	6	16	30	[38]
4	EF, exposure frequency	day/year	350	350	350	350	[37]
5	BW, body weight	kg	11.4	18.6	56.8	80	[38]
6	AT, average exposure time	day	365	2190	5840	10,950	[38]
7	RfD, reference dose	mg/kg day	1.6	1.6	1.6	1.6	[16]

**Table 2:** Descriptive statistics of physicochemical variables for drinking water samples (Min—minimum, Max—maximum, SD—standard deviation, KZ MPL—Kazakhstani maximum permissible limit, WHO MPL—[11] maximum permissible limit).

Samples *n* = 80	pH	EC (µS/cm)	TDS (mg/L)	Ca^2+^ (mg/L)	Mg^2+^ (mg/L)	K^+^ (mg/L)	Na^+^ (mg/L)	NO_3_^−^ (mg/L)	SO_4_^2−^ (mg/L)	Cl^−^ (mg/L)
Min	6.03	62	31	1.737	0.5	0.65	2.0	2.23	6.82	2.78
Max	8.25	993	497	99.58	18.34	2.86	104.8	59.8	211.4	58.8
Mean ± SD	7.42 ± 0.40	396.7 ± 179.2	202.7 ± 88.1	42.57 ± 21.49	8.01 ± 6.96	1.54 ± 0.57	11.49 ± 17.37	16.5 ± 13.0	29.73 ± 50.63	9.85 ± 8.68
KZ MPL Limit	6–9	-	1000	-	-	-	200	45	-	350
WHO MPL	6.5–8.5	-	600	-	-	-	-	50	-	-

**Table 3:** Hazard quotients (HQs) for different age ranges due to the consumption of nitrate in drinking water (underlined HQs indicate health risk).

Sample No.	District	HQ Adult	HQ Teen	HQ Child	HQ Infant
13	Alatau	0.60	0.68	0.81	1.04
18	Alatau	0.60	0.67	0.80	1.04
20	Alatau	0.64	0.72	0.86	1.12
24	Zhetisu	0.61	0.68	0.81	1.06
30	Zhetisu	0.65	0.73	0.87	1.12
32	Auezov	0.68	0.76	0.91	1.18
33	Auezov	0.80	0.90	1.08	1.40
35	Auezov	1.12	1.26	1.50	1.95
37	Auezov	0.59	0.67	0.79	1.03
38	Auezov	1.07	1.21	1.44	1.86
39	Auezov	0.68	0.77	0.92	1.19
43	Nauryzbay	0.77	0.87	1.03	1.34
Minimum		0.04	0.05	0.06	0.07
Maximum		1.12	1.26	1.50	1.95
Mean		0.31	0.35	0.41	0.54

## Data Availability

The data presented in this study are available on request from the authors.
